# Quantifying Plant Viruses: Evolution from Bioassay to Infectivity Dilution Curves along the Model of Tobamoviruses

**DOI:** 10.3390/v16030440

**Published:** 2024-03-12

**Authors:** Shaheen Nourinejhad Zarghani, Mehran Monavari, Amin Nourinejhad Zarghani, Sahar Nouri, Jens Ehlers, Joachim Hamacher, Martina Bandte, Carmen Büttner

**Affiliations:** 1 Division Phytomedicine, Faculty of Life Sciences, Albrecht Daniel Thaer-Institute of Agricultural and Horticultural Sciences, Humboldt-Universität in Berlin, Lentzeallee 55–57, 14197 Berlin, Germany; sahar.nouri@hu-berlin.de (S.N.); ehlerjen@hu-berlin.de (J.E.); martina.bandte@agrar.hu-berlin.de (M.B.); carmen.buettner@agrar.hu-berlin.de (C.B.); 2 Section eScience, Federal Institute for Materials Research and Testing, Unter den Eichen 87, 12205 Berlin, Germany; mehran.monavari@bam.de; 3 School of Mechanical Engineering, Hamburg University of Technology, Eissendorfer Str. 38, 21073 Hamburg, Germany; amin.nourinejhad.zarghani@tuhh.de; 4 Menno Chemie Vertrieb GmbH, Langer Kamp 104, 22850 Norderstedt, Germany; 5 Institute of Crop Science and Resource Conservation (INRES)—Plant Pathology, Universität Bonn, Nussallee 9, 53115 Bonn, Germany; hamacher@uni-bonn.de

**Keywords:** local lesion, qRT-PCR, *Nicotiana*, ToBRFV, TMV, CGMMV, disinfection, modeling

## Abstract

This review describes the development of the bioassay as a means of quantifying plant viruses, with particular attention to tobamovirus. It delves into various models used to establish a correlation between virus particle concentration and the number of induced local lesions (the infectivity dilution curve), including the Poisson, Furumoto and Mickey, Kleczkowski, Growth curve, and modified Poisson models. The parameters of each model are described, and their application or performance in the context of the tobacco mosaic virus is explored. This overview highlights the enduring value of the infectivity dilution curve in tobamovirus quantification, providing valuable insights for researchers or practitioners of bioassays and theoreticians of modeling.

## 1. Introduction

Bioassays have played an important role for decades as a key tool in plant virology. Despite technological advancements, their relevance has remained strong in diagnostic laboratories until now as they allow the detection of incompletely characterized viruses and viroids or for which specific serological and molecular tests are unavailable. These assays rely on the proven ability of viruses and viroids to spread from infected to healthy plants through mechanisms such as mechanical inoculation, transmission by natural vectors like insects, mites, fungi, or free-living nematodes, and grafting [1]. Although mechanical inoculation is the most commonly used method due to its simplicity, it is only applicable to viruses that can be mechanically transmitted, such as tobamoviruses [2]. Mechanical inoculation involves the introduction of infectious sap, virus particles, or viral RNA by rubbing them onto the surface of a plant leaf. After the entry of the virus into plant cells, several scenarios could unfold. They have been reviewed and updated by increasing our understanding of plant–virus interactions [3,4,5,6,7].

Local lesions are small spots that appear on the leaves of plants infected with some viruses. Different types of local lesions are known: inter alia, necrotic, chlorotic, and starch lesions (after iodine staining) [8]. For the first time, in 1929, Francis O. Holmes noticed that local lesions could appear after the mechanical inoculation of *Nicotiana* spp. with tobacco mosaic virus (TMV) variants, so he developed the mechanical inoculation assay [9]. The procedure of mechanical inoculation and different factors that might increase the success of inoculation have been described [10,11,12]. Furthermore, the local lesion phenomenon and its role in plant resistance have been reviewed [8,13]. Additionally, Holmes showed the correlation between the number of local lesions and the concentration of the virus [9]. That is because the number of lesions is directly proportional to the amount of virus applied to the leaf. Since then, the quantification of the virus titer via local lesion assay or how virus concentration relates to local lesion development has attracted attention. As a result, scientists have developed various models to accurately measure virus concentrations using local lesions [14,15,16,17,18,19,20].

The Poisson-based models, particularly those developed by Bald, take inspiration from the concept of one particle-one region, assuming that a local lesion occurs when at least one virus particle is present in the inoculum and inoculated onto a host leaf surface. To account for the unpredictable spread of virus particles within susceptible regions, the Poisson distribution is utilized [14,15]. Building upon this idea, Furumoto and Mickey introduced their hypermetric model, which posits that each virus particle has an equal opportunity to infect a cell, although this likelihood may differ among cells [17,18].

On the other hand, the logistic or Growth curve model offers a versatile approach, with minimum assumptions needed about the experimental system. Gokhale and Bald developed a logistic curve that describes the relationship between the expected proportion of infected regions and the logarithm of virus concentration [19].

Kleczkowski introduced a unique approach by assuming that susceptible regions on a leaf vary in their susceptibility, challenging the one particle-one region hypothesis. His model, based on the normal distribution of minimum effective dosage, considers the variability in susceptibility among different regions of the leaf surface [16].

In this review, we describe and compare the available models for the infectivity dilution curves via bioassay by focusing on tobamovirus. The story is through the analysis of basic models for the correlation between the concentration of virus particles and the number of local lesions (the infectivity dilution curve). The initial parameters of the Poisson model, the Furumoto and Mickey models, the Kleczkowski model, the Growth curve model, and the modified Poisson model are described and explored in terms of their application or performance in the context of the tobacco mosaic virus. We also summarized the design of the infectivity dilution curve experiments in the greenhouse.

## 2. Models for Correlation of Local Lesion Host and Virus Concentration

### 2.1. Poisson-Based Model and Its Variations in Tobamovirus Quantification

The fundamental theory of these models is based on the one particle-one region concept. This theory suggests that a local lesion occurs when at least one virus particle is present in the inoculum and is appropriately introduced into susceptible host tissue. It is similar to how the growth or absence of bacteria depends on the presence or absence of at least one viable bacterium in the fluid volume inoculated on a suitable medium. In this theory, there is no consideration for variations in susceptibility among different regions of the leaf surface of the local lesion host. Therefore, it is presumed that the susceptible regions (entry points of virus particles) of a leaf surface of the local lesion host have the same chance of becoming infected, and the distribution of particles inside the regions follows the Poisson distribution [14]. The first model of Bald used the same assumption as Youden [21]. The probability of causing all infections depends on the average number of virus particles inside the susceptible region, and this number is directly proportional to the virus concentration in the inoculum or virus suspension. This theory does not allow for any variation in the susceptibility between different susceptible regions of the host since it assigns the same chance for the entry points [16]. For TMV, Youden [21] proposed the following formula:

(1) $$Y=N_{max}\;(1-e^{-ax})$$

where Y is the number of lesions produced at a relative concentration *x* of the virus suspension, $N_{max}$ is the maximum number of lesions obtainable, and *a* is a constant. Due to the technical materials, the error was statistically meaningful (using the *Chi*-squared test) and known. So, Bald [14] corrected the technical process and modified the formula as follows:

(2) $$Y=N_{max}\;(1-e^{-pn_{1}x})$$

where the parameter *a* in the Youden formula changed to $pn_{1}$ where p is the chance of a single particle entering to cause infection, *n* is the number of possibly infective particles, and $n_{1}$ is the value of *n* for an undiluted inoculum. This formula has three uncontrolled parameters: $N_{max}$ , *p*, and $n_{1}$ . The parameter $N_{max}$ can be easily obtained after the inoculation experiment, and it is the highest number of local lesions observed on the half-leaf units. From the point of implementation of the models, there are no differences between Formulas (1) and (2) since parameters *a* or $pn_{1}$ have the same value. It should be mentioned that the parameter *a* can be calculated via $a=-\frac{ln\;\left(1-\frac{Y}{N_{max}}\right)}{x}$ . For example, if the number of observed local lesions in a serially diluted inoculum with the dilution factor of 2 (i.e., 1/2, 1/4, 1/8, 1/16, 1/32, 1/64, 1/128, and 1/256) were 138.8, 104.6, 64.0, 39.0, 24.2, 10.6, 4.0, and 2.4, and the maximum number of observed lesions in the half-leaf units was 160 ( $N_{max}=160)$ , then the $pn_{1}$ for the mentioned dilution range of 1/2, 1/4, 1/8 and so on can be calculated as a mean of 4.042, 4.242, 4.087, 4.470, 5.248, 4.387, 3.241, and 3.869, which is equal to 4.198.

Furumoto and Mickey [17,18] developed a hypermetric model based on the basic assumption of the Bald model, the one particle-one region assumption. They supposed that since the one particle-one region model has proven effective in studying bacterial and animal virus systems, a similar mode of infection might apply to plant virus systems. As a result, the original hypothesis of one particle per region was revised to account for inconsistencies between the proposed model and real-world findings. This revision was made based on two fundamental assumptions. According to the first assumption, a single “region” is equivalent to a single cell on the surface. It means that the parameter *N* represents the total number of susceptible cells per area abraded and could be very large, possibly reaching up to 1,000,000 for the entire leaf. The second assumption proposes that each virus particle has an equal chance of penetrating and infecting a cell, but this may vary between cells due to various factors, such as rubbing. Another way to express this assumption is to say that each cell has a unique probability distribution for being infected by a specific virus particle. The developed model of the one particle-one region hypothesis addresses the notion that infection probability may vary between individual cells and can be better represented through the use of a probability distribution. By defining each individual epidermal cell as its own region, any confusion surrounding the definition of a region is eliminated. Moreover, the use of a probability distribution to describe cell infection probabilities allows for flexibility in experimental determination. While this approach may not fully determine the infectivity dilution curve, its lack of rigid specificity is not seen as a disadvantage. It is believed that a universal curve does not apply to all experiments. 

The revised hypothesis suggests that a single virus particle can initiate an infection if it is introduced into a suitable cell. Although the experimental data alone cannot fully support this idea, the fact that they align with the results obtained lends credence to this hypothesis. Furthermore, the results provide valuable descriptive information and offer intriguing observations about the interactivity dilution curve, even if they do not completely reject alternate models, such as the one particle-one region model. The modified one particle-one region by Furumoto and Mickey was defined as (i) epidermal cells vary in their susceptibility to the infection; (ii) virus particles—*k* in number—available outside a cell have a Poisson distribution with mean, *ɑ*, proportional to concentration; (iii) the number of virus particles entering the cell with a fixed probability *x* of penetration has a binomial distribution with parameters *k* and *x*; and (iv) that *x* has a *β* distribution with parameters *α* and *β*. They reached the following relationship:

(3) $$Y=N_{FM}\alpha (1+cV/\beta )\;\mathrm{Furumoto}\;\mathrm{and}\;\mathrm{Mickey}\;\mathrm{Model}\;I$$

where *Y* is the expected number of lesions per half-leaf, $N_{FM}$ is the total number of susceptible regions, *c* is the constant, *V* is the virus concentration in the inoculum, and *α* and *β* are constants determining the distribution of cell penetration probabilities. This equation has two parameters, the values of which are unknown and have to be adjusted to give the best possible fit to the experimental data. It is evident that the parameters $N_{FM}$ and α, as well as *c* and *β*, are not identifiable separately. At that time, this hypermetric function was too complicated to use for fitting the data, unless $N_{FM}$ was fixed to 400,000. This number comes from the microscopic examinations of the leaf surface of *N. glutinosa*. They reported that, on average, there are 20 cm^2^ of leaf surface per half-leaf. Thus, there are roughly 400,000 epidermal cells on the upper surface of each half-leaf. Even with these changes, the model did not fit the data; therefore, the authors simplified the modified equation to Model II as follows:

(4) $$Y/N_{FM}=1-e^{-cV}\;\mathrm{Furumoto}\;\mathrm{and}\;\mathrm{Mickey}\;\mathrm{Model}\;\mathrm{II}$$

which is the same model described by Bald [14] with a different definition of *N*. Here, $N_{FM}$ is a hypothesized (not observed) number, which is usually estimated by inspection and extrapolation as the limit of lesions that appear at high concentrations of inoculum. *Y/* $N_{FM}$ is the expected proportion of infected regions in a total $N_{FM}$ of susceptible regions against the logarithm of virus concentration. Hence, the Bald model [14] can be assumed as a special case and was not investigated separately.

In 1990, a modified Poisson model was introduced [15] to see if it fits best with all infectivity dilution curves. This modified Poisson model uses the Furumoto and Mickey Model II to estimate c values ( $\tilde{c}$ ) for each observation which, in turn, is used to estimate *Y* ( $\hat{Y}$ ), which is the expected number of lesions out of a total possible number $N_{MP}$ . To optimize this model, for each i, ${\tilde{c}}_{i}V_{i}$ is calculated from $\hat{Y_{i}}=N_{MP}\;(1-e^{-{\tilde{C}}_{i}V_{i}}$ *)*. In the next step, the $log\left({\tilde{c}}_{i}V_{i}\right)$ is regressed against the $log\left(cV_{i}\right)=logm_{i}$ , logs of the observed concentrations, to obtain $log({\tilde{c}}_{i}V_{i})=a+blog\left(m_{i}\right)$ . These estimated parameters are used in the main equation

(5) $${Ŷ}_{i}=N_{MP}\left(1-e^{-{Am}_{i}^{b}}\right)$$

where $A=10^{a}$ .

### 2.2. Logistic Model or Growth Curve Model in Tobamovirus Quantification

The logistic or the Growth curve model was also introduced for correlating the number of local lesions and concentration of virus suspensions. Unlike previous models, this method requires minimal assumptions about the experimental system and is applicable to a broad range of scenarios. Previous formulations for single-component viruses, based on different assumptions, have hindered the development of a unified quantitative theory. These formulations, derived from the Poisson series and confluent hypergeometric function, presented challenges in fitting infectivity dilution curves, particularly for common TMV. The Growth curve model defines a logistic curve relating the expected proportion of infected regions (*Y/N*) to the logarithm of concentration ( $t={log}_{10}\;V$ ). The model exhibits simplicity and is akin to the Poisson model at low-to-moderate concentrations, transitioning to an asymptotic behavior at higher concentrations. The limiting concentration is defined as 100% virus in the hydrated state, and the reduction in predicted values at this concentration is attributed to mutual interference between closely packed virus particles. In 1987, Gokhale and Bald [19] derived the relationship between the expected proportion of infected regions measured by *p* = *Y/N* and the logarithm of virus concentration ( $t={log}_{10}\;V$ ) as a logistic model. In this model, they assumed that the rate of increase in *p* as a function of *t* is proportional to *p* itself and its deficiency ( 1−p ), i.e., (d*p/*d*t*) $\;\propto p\;\left(1-p\right)$ . The resulting equation becomes:

(6) $$p=\frac{Y}{N_{L}}=\frac{1}{1+\beta \;e^{-\gamma t}}$$

where *β* and γ are positive constants of the model. The parameter $N_{L}$ , representing the total number of lesion sites available on a leaf, is estimated by minimizing the Pearson *Χ*^2^ statistics. The fitting procedure provides an objective approach without relying on arbitrary parameter values. It should be mentioned that the Growth curve model resembles the Poisson model at high concentrations of virus since $\beta \;e^{-\gamma t}$ is close to 0, and it represents the Poisson model. When the virus concentration is 0, t approaches −∞ ; thus Y=0 , meaning no lesion. At the higher concentrations toward a limit, it has an “asymptote” of *N*. 

### 2.3. Normal Distribution-Based Approaches in Tobamovirus Quantification

Kleczkowski (1950) introduced a model that basically differs from one particle-one region-based models by assuming that regions of a leaf vary in susceptibility and that different minimal amounts of virus are necessary to infect them [16]. He explained that there are two theories regarding the concentration of a virus in an inoculum and the appearance of local lesions in the host. One theory suggests that a local lesion develops as a result of the presence of at least one virus particle in the inoculum that has been suitably introduced into susceptible host tissue. This theory is analogous to the spread of a solution that might harbor bacteria on a suitable culturing medium. Therefore, the presence or absence of bacterial growth depends on the presence or absence of at least one viable bacterium in that solution inoculated upon a suitable medium. According to this idea, there is no consideration for differences in susceptibility between various parts of the plant leaf. Any differences would only come from changes in the number of susceptible areas. 

According to the other theory, susceptible regions on the leaf surface vary in susceptibility so that the minimum virus concentration necessary to initiate local lesions varies from one region to another [16].

Kleczkowski [16] used different ranges of the virus suspensions from low (max. 0.2 mg/mL) to high (20 mg/mL) concentrations with different dilution factors (2, 3.16, 4, and 5) to test the fitness of the different models in both low and high concentrations of the virus in the inoculum to requite different minimum virus concentrations to initiate local infection. He also tested the original models and assumptions using statistical analysis, graphical fitting of experimental data to different equations, and comparisons of experimental and computed values. In the end, his optimized model [16] is based on the assumption that regional susceptibility (e.g., leaf surface) varies in such a way that the logarithm of minimal effective dosage necessary to cause the formation of the lesion is normally distributed. In this model, the equation is as follows:

(7) $$Y=\frac{N_{K}}{\lambda \sqrt{2\pi }\;}\;\int_{-\infty }^{t}exp\;\left\{-\frac{1}{2}\;{\left(\frac{t-\xi }{\lambda }\right)}^{2}\right\}\;dt$$

where *Y* is the expected number of lesions per half-leaf, $N_{K}$ is the mean number of “susceptible regions” per half-leaf (which is a hypothesized number), *t* is the logarithm of virus concentration in the inoculum or dilution of infective sap, $\xi ={log\;x}_{0}$ , $x_{0}=$ virus concentration or dilution of infective sap when 50% of the susceptible regions develop lesions, and *λ* is the standard deviation. The parameters $\;N_{K}$ , *ξ*, and *λ* are unknown and have to be adjusted in a way that the model fits the experimental data. 

Equations introduced by Furumoto and Mickey’s hypothesis [17,18] have been compared with the hypothesis of Kleczkowski and vice versa [22]. The latter author explained that Furumoto and Mickey proposed three key assumptions: (1) a sole or single virus particle can trigger a local lesion after entering into a susceptible region of the host tissue; (2) every epidermal cell is a susceptible region; and (3) the probability of virus particle penetration differs among cells. When Kleczkowski was developing the model, the author had already tested the hypothesis that incorporated the assumption that single virus particles can develop a local lesion and, similarly, the probability of the penetration of virus particles varying between cells (i.e., Items 1 and 3), and Kleczkowski found that it aligned with experimentally obtained infectivity dilution curves. Furumoto and Mickey, however, misunderstood this conclusion, thinking it rejected Assumption (1). The author provided additional experimental evidence supporting Assumption (1) and a condition analogous to (3), indicating that the probability of successful infection initiation varies among different regions. Kleczkowski further explained that he also evaluated different hypotheses and conditions. Kleczkowski tested two hypotheses: (A) assumes all susceptible regions are identical, and (B) assumes a dose of virus particles is needed to start infection, and susceptibility varies with logarithms of minimal infection doses being normally distributed. He showed that Hypothesis (A) was false, while Hypothesis (B) seemed correct. However, Assumption (1) was not definitively rejected; instead, it was shown that if Assumption (4) (all susceptible regions are identical) is false, Assumption (1) might still be correct. It has been demonstrated that a single bacteriophage particle, when placed in a bacterial culture, can multiply and cause infection, but the probability varies with the age of the culture. This supports the idea that local lesions can be initiated by single virus particles in susceptible regions with varying probabilities [22,23].

### 2.4. Summarizing Key Factors or Assumptions Shaping Tobamovirus Quantification Models

When the developments of all introduced models are compared, they share or differ in a few key basic assumptions that can be summarized as follows: (a) a single virus particle that is introduced into a susceptible region of the host tissue can start infection and a local lesion; (b) each epidermal cell is a susceptible region; (c) the probability of virus particle penetration into the cells varies; (d) all susceptible regions are identical; (e) a minimum dose of virus particles is needed to initiate an infection in a susceptible region; and (f) susceptibility of the regions varies in such a way that the logarithms of minimal infective doses are normally distributed. 

Based on these assumptions, the Bald 1936 model follows Assumptions (a), (b), and (d), the Furumoto and Micky models are based on Assumptions (a), (b), and (c), and the Kleczkowski model is based on Assumptions (e) and (f). In contrast, in the Growth curve model, there are no clear explicit physical Assumptions (e) and (f). It should be mentioned that the Growth curve model is a mainly data-based model and has been used accordingly for modeling disease development or for describing bacterial Growth curves. 

### 2.5. Exploring Nonparametric Approaches for Comparisons in Local Lesion Test Assays

It should be mentioned that nonparametric methods have also been used for evaluating the half-leaf local lesion assays, using the direct number of the local lesions of transformed ones based on the equation suggested by Kleczkowski. It has been shown that the main difference between the parametric and nonparametric methods was reflected in the spread of the confidence limits calculated according to each. For example, depending on the sample size, it may be estimated that nearly 30 half-leaf units per dilution would be required in an assay evaluated by the nonparametric method to assure the same degree of precision obtained by the parametric method with only twelve leaves. This shows that parametric methods need at least 60% less work and space in the lab and greenhouse. For more data, readers are invited to refer to [20]. 

## 3. Evaluating Goodness of Fit for Various Models Applied to Experimental Data

**Figure 1:**
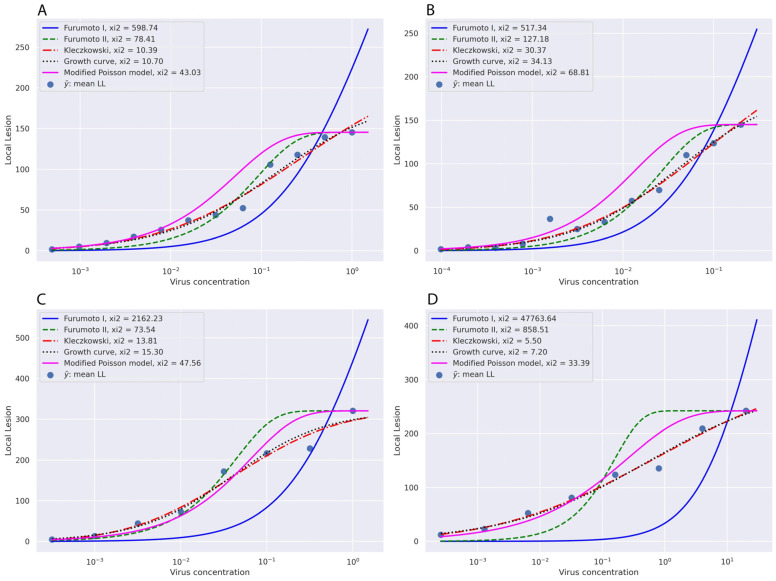
The estimated infectivity dilution curves according to the different models calculated based on the mean number of the local lesions on *N. glutinosa* as a local lesion host against the logarithm of virus concentrations in serially diluted virus suspensions. The raw data from Experiment No. 1 (**A**), Experiment No. 2 (**B**), Experiment No. 10 (**C**), and Experiment No. 13 (**D**) of Kleczkowski [16] were used for implementing the Kleczkowski model [16], Furumoto and Mickey Models I and II [17,18], the Growth curve model [19], and the modified Poisson model [15].

To compare which of the explained models best fits the experimental data, we utilized data from Kleczkowski’s experiments [16], some of which were also employed by other model developers [15,17,18,19]. As mentioned before, Kleczkowski used different concentrations, and we presented Experiment No. 1 with an initial concentration of 10 mg/mL ( ic=1 mg/mL) , dilution factor of 2 (f=2), and 24 replications (r=24) ; Experiment No. 2 with ic=0.2 mg/mL, f=2, and r=24 ; Experiment No. 10 with ic=1 mg/mL, f=3.16, and r=24 ; and Experiment No. 13 with ic=20 mg/mL, f=5, and r=12 . It should be mentioned that in Experiment No. 1, *N. glutinosa* plants were inoculated with tomato bushy stunt virus (ToBSV), while, in the remaining experiments, *N. glutinosa* plants were inoculated with TMV. These datasets were employed in various analyses following the method outlined in [24,25]. Figure 1 shows the graphical goodness of fit of different models with the experimental data. 

Based on the graphs, the Kleczkowski model and then the Growth curve model were fitted the best to all experimental data followed by the modified Poisson model. The Furumoto and Mickey Models I and II could not fit in most experimental data. The predicted values for the mean number of local lesions based on different models are presented in Table 1, Table 2, Table 3 and Table 4. 

By comparing these data, it can be understood that, in most cases, the Kleczkowski model and Growth curve model show the best estimation in comparison with the other models in both low and high concentrations of the viruses. Choosing a lower dilution factor increases the number of dilution points, enhancing model accuracy. It has been suggested to have at least six dilutions per experiment [15]. 

## 4. Machine Learning and Artificial Intelligence in Quantification of Viruses Using Local Lesions

### 4.1. Integration of Machine Learning Techniques in Local Lesion Assay Models

The recent advancements in the field of machine learning (ML) offer a compelling opportunity to enhance the measurement of plant viruses, especially with the integration of local lesion assays. By utilizing classical ML techniques, predictive models can now consider various influential factors, such as leaf age, environmental conditions (such as temperature and humidity), and the overall physiological state of the plant. These elements play a crucial role in determining the host’s susceptibility to viral infection and the resulting development of local lesions [12,24,26,27].

A solution for addressing the discrete nature of local lesion counts is using count-based models, such as generalized Poisson regression or negative binomial regression. Specifically, the negative binomial model, with its ability to adjust for over-dispersion, a common issue in count data where the variance exceeds the mean, provides a more reliable framework for analyzing count data in plant pathology, offering not only precise predictions but also insights into the data’s underlying distribution [28,29]. By being trained on a diverse dataset consisting of virus concentrations, environmental factors, and lesion counts, these ML models can accurately predict the virus concentration in an unknown sample while still being interpretable. 

### 4.2. Application of Deep Learning for Image-Based Disease and Virus Classification and Quantification

The integration of deep learning technologies in plant pathology represents a significant leap forward in detecting and classifying plant diseases. Recent comprehensive reviews such as the works of [30,31] underscore the transformative impact of deep learning methods over traditional approaches, emphasizing the sophistication in classification, detection, and segmentation capabilities that these technologies bring to the field of plant disease identification. Techniques such as supervised convolutional neural networks (CNNs) [32] and their variations, like faster region-based convolutional neural networks (RCNNs) [33] and SSD [34], have been effectively used for real-time disease detection in various plants. The use of generative adversarial networks (GANs) for unsupervised learning further enhances the efficiency of disease identification without the need for manual feature selection [35,36]. 

However, these deep learning models mostly focus on disease detection and classification, and their full potential for quantitative analysis of virus concentration in lesion assays remains largely unexplored. 

## 5. Application of Infectivity Dilution Curve in Virucidal Efficacy of Disinfectants

Various methods are available for the quantification of plant viruses relying on virus genome, proteins, and pathogenicity, as well as physico-chemical properties. The real-time reverse transcription polymerase chain reaction (real-time RT-PCR) stands out as the predominant technique for both absolute and relative quantification of viruses [37,38], followed by the quantitative enzyme-linked immunosorbent assay (qELISA) [39,40]. In principle, real-time RT-PCR or digital PCR has the capability to detect even a single copy of viral genomic RNA or DNA, regardless of whether it is intact or aberrant. However, they do not provide information on the infectivity of these molecular components. As a result, bioassay remains the sole method at our disposal for evaluating infectivity, making it especially valuable when the primary focus is to evaluate virus infectiosity.

One of the greatest potentials of this method, as an example of its practical use, is in determining the virucidal efficacy of disinfectants and other treatments aimed at inactivating viruses. The availability of the infectivity dilution curve models for quantification of the viruses via bioassay allows the assessment of the virucidal efficacy of disinfectants [25]. 

The disinfection of greenhouses to eradicate phytopathogenic viruses has a long history and has already been studied for nearly 100 years [41,42]. Species of the tobamovirus genus have always been of great importance as they are highly stable. The type species TMV can remain infectious in dried-leaf material and non-sterile plant extracts at room temperature for at least 50 years [43]. TMV was found in cigarettes, although leaves undergo various processes [44] and even in the saliva of smokers [45]. It can withstand extreme pH levels, ranging from 3.0 to 9, and can even endure exposure to high temperatures of up to 90 °C when in sap form and 80 °C in its dry form [46,47,48].

This remarkable resilience to environmental factors and various agents presents a formidable challenge in terms of control. Moreover, the rapid and efficient transmission of viruses between plants worsens the economic repercussions for the agricultural industry—a defining trait of the tobamovirus group. The high capability of mechanical transmission of these viruses is such that a low percentage of them is sufficient to infect the entire greenhouse [49,50,51]. Tobamoviruses are not only transmitted through plant-to-plant contact but also during common horticultural activities, like pruning, irrigation, and harvesting. In addition, these viruses can also cling onto inanimate surfaces like glass, plastic, rubber, and metal, where they can stay infectious for a long time and spread to other production sites if proper cleaning and disinfection measures are not implemented [52]. Hygiene management, consisting of cleaning and disinfection, is an essential component in protecting crops in greenhouses from harmful organisms, such as tobamoviruses. However, the effectiveness of a disinfectant, in general, depends on many factors, such as the contact time [53,54], temperature, water quality (in particular, pH values and water hardness), application technique, and so on. A comprehensive illustration of the influencing factors can be found in [55]. 

In the past, numerous investigations addressed the efficacy of disinfectants to inactivate tobamoviruses. These experiments are of limited comparability as different inocula, disinfectants, and evaluation methods were applied (Table 5). As shown in this table, the efficacies of the tested disinfectants are hardly comparable when the initial concentration of tobamoviruses in a sample and the methods used for evaluating disinfection efficacy differ widely. In this regard, for future disinfection studies, it is imperative to use standardized and validated methods for quantifying virus concentration before and after disinfection. This will ensure the consistency and comparability of results.

Furthermore, previous studies were not able to prove the log depletion of infectious virus particles after application, but instead, the percentage of infected plants in relation to non-infected plants was often used as a basis for evaluating the disinfection efficacy. The method for evaluating the log depletion of infectious tobamoviruses using local lesion-inducing host plants is available [24,25]. By knowing the log depletion of the virus concentration through the application of disinfectants and the usual virus concentrations on contaminated surfaces in infested greenhouses, we will also be able to determine the intensity of disinfection measures for reliable tobamovirus inactivation in greenhouses in the future.

In order to make the methodology more widely applicable, a description of the approach is given below. It should be mentioned that the local lesion assay could be applied for the viruses that have a local lesion host. It is important to have access to the biological characteristics of the virus isolate and be sure about its purity and pathogenicity.

### 5.1. Preparation of the Standard Sample

The standard sample is the virus inoculum with determined virus particles. There are several general protocols for the purification of tobamoviruses [24,68,69]. Of course, the purified particles have to be intact and infectious particles (virions) that can be easily confirmed by electron microscopy and a simple inoculation test in a local or systemic host. The concentration of the purified particles can be measured by spectrophotometry, electron microscopy [70,71], ultrasensitive flow virometry [72], and viral quantitative capillary electrophoresis [73]. Kleczkowski showed that infectivity dilution curves can be successfully applied even when working with diluted plant sap. This is particularly useful when purified virus particles are not accessible or are challenging. However, it is important to maintain consistency in the approach by using the same inoculum throughout all experimental treatments. This ensures reliable and comparable results across different conditions or interventions.

**Figure 2:**
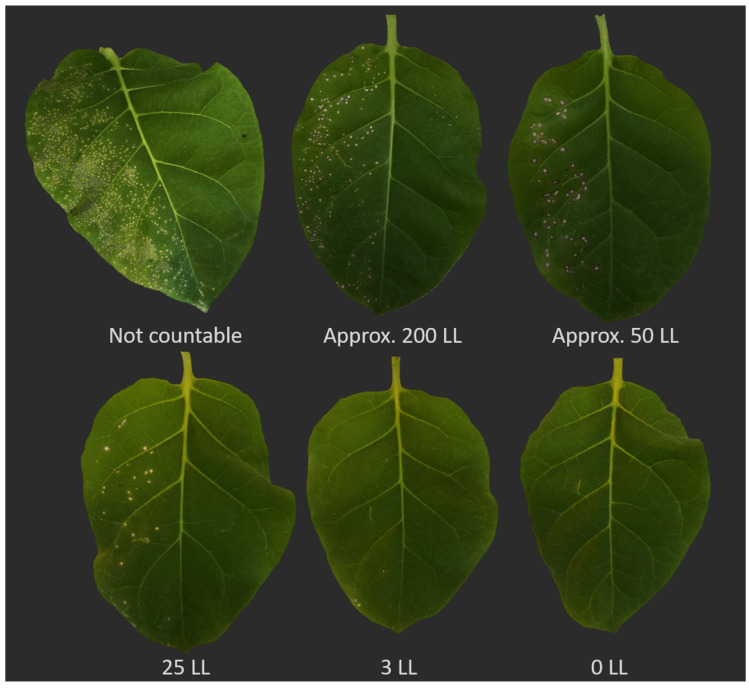
Optimizing virus concentration for accurate local lesion counting and adjusting concentration to prevent lesion aggregation. The range of virus concentration should be fine-tuned through preliminary tests to ensure that local lesions do not aggregate (top left), thereby avoiding the formation of a larger necrotic lesion. This adjustment enables easy and accurate lesion counting. LL: local lesion.

It is advisable to establish a preliminary inoculation series with varying higher virus concentrations (e.g., 20 mg/mL, 10 mg/mL, 5 mg/mL, and 2 mg/mL). This ensures obtaining the maximum concentration of virus particles, facilitating countable local lesions. The same principle applies to plant sap, which can be diluted with different initial ratios of the extraction buffer or water and various dilutions to estimate the resulting number of local lesions and virus concentration. This process offers insight into the obtained number of local lesions, particularly assessing whether the inoculum contains sufficient infectious particles for measurability after treatments like disinfection or thermotherapy. If the virus concentration is lower than the average concentration in nature, there is a risk of overestimating treatment efficacy. Hence, selecting the appropriate concentration is crucial to ensure accurate results. To minimize errors, it is essential to consider the reduction in infectivity due to dilution with the disinfectant volume. Moreover, the virus concentration in the inoculum should closely align with the natural virus concentration in the plant to avoid overestimating results. Our observations indicate that a maximum of about 250 local lesions on one half-leaf unit can be easily counted. However, when local lesions are very close to each other, they may merge, forming a large necrotic region on the leaf, making it impossible to count individual lesions (Figure 2).

Simultaneously, it is crucial to determine the minimum virus concentration capable of inducing lesions. Once the highest and lowest virus concentration ranges for the bioassay are established, create a series of virus dilutions with varying factors, such as 2, 5, and 10. This step is essential for fitting the data to construct the infectivity dilution curve at low inoculum concentrations. This becomes particularly important after treatments, allowing for the tracking of the low concentration of the virus or traces of virus load on different surfaces post-treatment. The serial dilution can be prepared in an inoculation buffer or the desired plant sap, for example, tomato leaves, in the case of ToBRFV, since tomato is the natural host.

Based on our experiences, higher concentrations of the virus should be used as an inoculum for disinfection treatments. Ideally, the selected higher virus concentrations should exceed the virus concentration in the systemic or natural host with severe symptoms. In comparison to the chosen lower virus concentration for disinfection assays, results with a higher virus concentration will be more reliable and closer to the real situation in the greenhouse. This concentration should be determined after preliminary experiments and checked after determining the virus concentration in a systemic host or main host. For instance, if the highest virus concentration of ToBRFV in a susceptible variety of tomatoes is 1 mg/mL, the virus concentration in the inoculum should be at least 1 mg/mL. Using a lower concentration or an inoculum with a lower virus concentration can lead to underestimating the virus concentration for virucidal efficacy. Therefore, starting with a higher concentration of the viral inoculum is logical to ensure reliability.

When conducting local lesion assays, it is imperative to minimize deviations and mistakes caused by various environmental factors. These can include factors such as light cycles, temperature, the species and age of the local lesion host, the position of the leaf on the plant, nutritional conditions, and the method of inoculation. It has been reported that keeping plants in darkness or low light conditions for 24 h before inoculation can increase the number of local lesions and affect their growth. Furthermore, the addition of substances like phosphate-based buffers or particles of carborundum or diatomaceous earth during inoculation has been shown to have a positive effect on local lesion formation [26]. Using the phosphate buffer as an inoculation buffer and abrasives is known to boost the amount of local lesions [27]. The inclusion of bentonite, which counteracts ribonuclease, results in an increase in the number of local lesions. This was observed with naked TMV RNA but not with intact virions [26]. When altering the source of inoculum, such as through various purification methods, it is advised to conduct ANOVA analysis on data collected from randomized block experiments to determine any significant variances in the number of lesions caused by different inocula. This guarantees minimal divergence between the inocula. It has been shown that the position of the leaf or half-leaves was not statistically significant and did not impact the number of local lesions induced by ToBRFV in *N. tabacum* cv. *Xanthi NN* and *N. glutinosa* [24].

### 5.2. Designing the Inoculation Experiment and Collecting the Data

**Figure 3:**
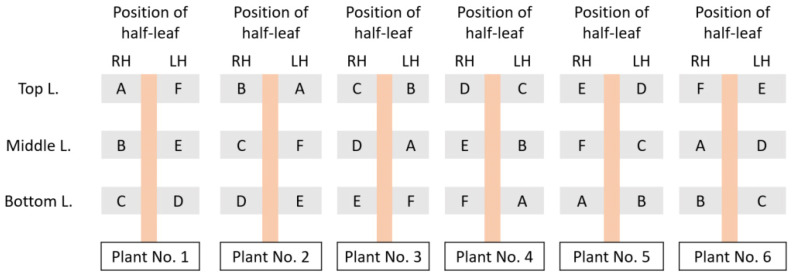
Example of randomization for six inoculum dilutions (A–F) using six half-leaf units from three well-grown leaves of a suitable local lesion host. “L.” denotes the leaf, “RH” and “LH” denote the right half-leaf unit and left half-leaf unit, respectively.

The inoculation for correlating the number of local lesions and the virus concentration was set up in half-leaf units using the Latin square design. Randomization is crucial not only between leaves but also between plants in a treatment group. There are several ways for randomization of the half-leaf units that have been reviewed, and examples are given depending on the type of local lesion host [12,16,74]. In the instance of *N. tabacum* cv. *Xanthi* NN, three well-developed leaves were employed for inoculation; nonetheless, in certain instances, four leaves of *Nicotiana* spp. plants were utilized for this purpose. It is imperative to exercise careful consideration in the selection of individuals serving as the local lesion hosts. The implementation of experimental homogeneity by employing plants of uniform size and identical phenological stages is recommended as a prudent approach to mitigate potential sources of error and reduce variability in the quantification of local lesions. Furthermore, it is advisable to explore the existence of any potential correlation between plant size, phenological stage, and the observed local lesion counts to enhance the rigor of the experimental design. To assess and ensure both the precision and accuracy of estimated virus concentrations, the inclusion of a spike control—comprising an additional sample with a known virus concentration—is recommended. It has been demonstrated that the local lesion assay achieves a satisfactory level of accuracy when implemented with 16 replications [20,75]. For the establishment of models, the developers used 24 half-leaf units for each dilution, and the experiments were repeated three times. In Figure 3, a schematic sample of the inoculation plan for 6 dilutions and 24 half-leaf units is shown. To label the half-leaf units, it is possible to use colored paper sticks on petioles. The number of local lesions can be counted three to several days after inoculation (depending on the virus and host combinations), and they are categorized based on the designed Latin square experiment. 

In 1934, Youden and Beale [21] were the first to emphasize the crucial role of statistical design and variance analysis in local lesion experiments. This is necessary because susceptibility can vary greatly among plant species and even within the leaves of the same plant. In the following years, scientists have employed a range of statistical techniques to analyze their findings. While some have directly applied these methods to lesion counts, others have utilized them on the logarithms of lesion numbers, recognizing the benefits of a more comprehensive approach. These methods assume that the variable follows a normal distribution and the variance is independent of the population mean. Therefore, Kleczkowski introduced the transformation method and examined the distribution of localized lesions, determining the most effective statistical approach for them [76,77]. 

The number of local lesions or the mean number of local lesions and the corresponding virus concentration can be used directly for estimating the model parameters except for in the Kleczkowski model [16]. The number of local lesions can be subjected to data transformation, and Kleczkowski suggested the following formula z=log⁡(x+c) (where *c* is a constant) when the mean value of the number of local lesions is greater than 10 since it is not suitable for lower numbers of local lesions. If this is the case, the following formula $z=log\frac{1}{2}[x+c+\surd (x^{2}+2cx)]$ has been suggested when the mean value of the number of local lesions is greater than 1.5 [16,77]. It should be mentioned that *c* is an arbitrary parameter and can be optimized for each experiment. Simply, it has been suggested to use the 5, 10, 15, and 20 as a *c* value, and then, test the best value [77]. Fry and Taylor [78] tested the formula z=log⁡(x+c) for its applicability in *N. glutinosa* L. challenged with TMV or tomato spotted wilt virus (ToSWV), *N. tabacum* L. var. White Burley inoculated with turnip mosaic virus (TuMV), and *Phaseolus vulgaris* L. var Black Valentine challenged with tobacco necrotic virus (TNV) with different numbers of trials. They found that the most reliable transformation for both half-leaf experiments on beans, tobacco, and *N. glutinosa*, as well as whole-leaf experiments on beans, is log (*x* + 12) [78].

## 6. Conclusions

We described the crucial factors and approaches necessary for precise virus measurement using local lesion assays. To obtain a more accurate quantification, it is crucial to consider variables such as environmental factors, host attributes, and inoculation methods. The importance of striking a delicate balance, ensuring that lesions are visible for counting but not merging with necrotic areas and choosing the right concentration of the initial inoculum, plays an important role in obtaining more reliable data. Among the established methods for correlating the virus concentration and the number of local lesions, the Kleczkowski model [16] and Growth curve model [19] prove to be the most suitable for fitting the majority of infectivity dilution curves of TMV, ToMV, and ToBRFV [15,16,19,24,25]. However, it is crucial to note that while these models excel in fitting curves, they do not provide insights into the underlying mechanisms and steps of the inoculation process. It should also be acknowledged that the parameters of each model, selected based on the involved factors or variables in the inoculation process, contribute to the overall understanding of local lesion dynamics. Therefore, the merger of machine learning and convolutional neural networks in correlation with the number of local lesions and the virus concentration in the inoculum for virus quantification ushers in a promising horizon in plant virology and more accurate quantification via bioassay. These advanced techniques pave the way for more precise, efficient, and scalable quantification, potentially revolutionizing virus research. Additionally, our investigation into infectivity dilution curves and their use in evaluating the effectiveness of disinfectants highlights the necessity of standardized methods. By employing local lesion assays, we gain a unique advantage in assessing infectivity and gathering insights to combat the resilient nature of tobamoviruses. We offer a practical guideline that serves as a thorough protocol for both researchers and practitioners. These necessary steps, such as establishing preliminary inoculation series, determining ideal concentrations, and implementing randomization techniques, are crucial for obtaining strong and trustworthy results. Using the quantification method based on the infectivity dilution curve, the depletion of infectious tobamovirus particles can now be determined after almost 100 years of research. This will contribute to more comparable results of disinfection studies in the future. However, other aspects of disinfectant testing, such as the application volume used, contact time, water quality, etc., must be standardized even more so that reliable and scientifically evaluated recommendations can be given to growers.

Overall, with this review, we aim to enrich the ongoing discussion about virus quantification and emphasize the continued importance of local lesion assays. We anticipate that our findings, based on technological advances and the fine-tuning of experimental methods, will advance the field and provide invaluable resources for researchers and agricultural experts alike.

## Figures and Tables

**Table 1:** The mean number of observed and predicted necrotic local lesions on *N. glutinosa* with serially diluted ToBSV in Kleczkowski’s Experiment No. 1 ( ic=1 mg/mL, f=2,r=24) [16].

Virus Concentration (mg/mL)	Mean No. of Observed LL *	Computed Y Based on				
		Kleczkowski Model	Furumoto and Mickey Model I	Furumoto and Mickey Model II	Growth Curve Model	Modified Poisson Model
1	145.5	153.9	150.7	145.5	151.5	147.9
0.5	139.3	132.8	130.0	144.8	134.5	137.1
0.25	117.9	110.5	109.5	135.7	113.6	117.1
0.125	105.8	88.4	89.4	107.9	90.5	91.8
0.0625	52.3	67.7	70.1	71.5	67.8	67.2
0.03125	44.0	49.5	52.0	41.7	48.0	46.7
0.015625	37.4	34.5	36.1	22.6	32.5	31.4
0.0078125	25.8	22.9	23.1	11.8	21.2	20.6
0.00390625	17.3	14.4	13.7	6.0	13.5	13.3
0.001953125	9.6	8.6	7.6	3.0	8.4	8.5
0.000976563	5.0	4.9	4.1	1.5	5.2	5.4
Model parameters **		$N_{K}$ : 225.48	$N_{FM}$ : 30.11	$N_{FM}$ : 145.46	$N_{L}$ : 188.46	$N_{MP}$ : 151.50
		λ: 1.21	C: 147.91	c/β: 10.82	β: 0.244	A: 2.23
		ξ: −0.57			γ: 1.65	b: 0.67
						c: 2.16
Error *X*^2^		10.4	12.3	78.4	10.7	10.3

* Local lesion. ** The parameters are explained in this manuscript and are summarized in [24,25].

**Table 2:** The mean number of observed and predicted necrotic local lesions on *N. glutinosa* with serially diluted TMV in Kleczkowski’s Experiment No. 2 ( ic=0.2 mg/mL, f=2,r=24) [16].

Virus Concentration (mg/mL)	Mean No. of Observed LL *	Computed Y Based on				
		Kleczkowski Model	Furumoto and Mickey Model I	Furumoto and Mickey Model II	Growth Curve Model	Modified Poisson Model
0.2	145.1	147.9	144.2	145.0	145.0	142.7
0.1	123.8	123.5	121.6	141.6	125.2	127.7
0.05	110.0	99.0	99.5	122.5	101.8	103.8
0.025	69.8	75.9	78.1	87.8	77.4	77.4
0.0125	57.4	55.6	58.1	53.9	55.2	54.1
0.00625	33.0	38.7	40.5	30.1	37.2	36.1
0.003125	25.0	25.6	26.1	15.9	24.0	23.3
0.0015625	36.6	16.1	15.5	8.2	15.0	14.8
0.00078125	8.0	9.6	8.7	4.2	9.2	9.3
0.000390625	3.4	5.4	4.6	2.1	5.6	5.8
0.000195313	4.0	2.9	2.4	1.0	3.4	3.6
0.0000977	1.8	1.4	1.2	0.5	2.0	2.2
Model parameters **		$N_{K}$ : 224.71	$N_{FM}$ : 33.14	$N_{FM}$ : 145.08	$N_{L}$ : 188.55	$N_{MP}$ : 149.09
		λ: 1.19	C: 382.55	c/β: 37.18	β: 0.09	A: 3.83
		ξ: −1.01			γ: 1.73	b: 0.7
						c: 3.8
Error *X*^2^		33.8	127.2	30.4	34.1	35.2

* Local lesion. ** The parameters are explained in the manuscript and are summarized in [24,25].

**Table 3:** The mean number of observed and predicted necrotic local lesions on *N. glutinosa* with serially diluted TMV in Experiment No. 10 with ic=1 mg/mL, f=3.16,r=12 by Kleczkowski [16].

Virus Concentration (mg/mL)	Mean No. of Observed LL *	Computed Y Based on				
		Kleczkowski Model	Furumoto and Mickey Model I	Furumoto and Mickey Model II	Growth Curve Model	Modified Poisson Model
1	320.7	296.9	320.7	320.7	298.7	317.8
0.316	228.9	263.8	258.1	320.4	270.3	287.3
0.1001	216.9	210.8	196.2	288.0	217.8	212.7
0.0316	171.9	145.6	136.5	165.0	146.1	130.7
0.01002	72.3	84.1	82.5	65.5	79.6	71.4
0.00317	44.1	39.6	41.0	22.4	37.0	36.6
0.001004	13.3	15.0	16.5	7.3	15.7	18.2
0.000317	4.8	4.5	5.8	2.3	6.4	8.9
Model parameters **		$N_{K}$ : 320.67	$N_{FM}$ : 54.68	$N_{FM}$ : 320.67	$N_{L}$ : 320.67	$N_{MP}$ : 320.67
		λ: 0.96	C: 351.47	c/β: 22.79	β: 0.074	A: 2.60
		ξ: −1.39			γ: 1.86	b: 0.64
						c: 2.53
Error *X*^2^		13.8	16.9	73.5	15.3	29.6

* Local lesion. ** The parameters are explained in the manuscript and are summarized in [24,25].

**Table 4:** The mean number of observed and predicted necrotic local lesions on *N. glutinosa* with serially diluted TMV in Experiment No. 13 with c=20 mg/mL, f=5,n=12 by Kleczkowski [16].

Virus Concentration (mg/mL)	Mean No. of Observed LL *	Computed Y Based on				
		Kleczkowski Model	Furumoto and Mickey Model I	Furumoto and Mickey Model II	Growth Curve Model	Modified Poisson Model
20	242.2	238.9	228.6	242.2	235.9	236.8
4	209.2	200.3	192.9	242.2	202.0	205.7
0.8	135.5	157.2	157.3	240.6	159.2	158.5
0.16	123.7	114.4	121.8	154.0	114.0	110.3
0.032	81.0	76.5	86.5	44.3	74.5	71.5
0.0064	52.3	46.8	52.6	9.6	45.2	44.3
0.00128	23.0	26.0	24.0	2.0	26.0	26.6
0.000256	12.2	13.1	7.3	0.4	14.5	15.8
Model parameters **		$N_{K}$ : 319.03	$N_{MP}$ : 22.13	$N_{MP}$ : 242.17	$N_{L}$ : 292.20	$N_{MP}$ : 248.74
		λ: 2.03	C: 1527.89	c/β: 6.32	β: 0.766	A: 1.08
		ξ: −0.06			γ: 0.9	b: 0.34
						c: 1.04
Error *X*^2^		5.52	8.86	858.51	7.20	9.19

* Local lesion. ** The parameters are explained in the manuscript and are summarized in [24,25].

**Table 5:** Overview of bioassays used to determine the efficacy of disinfectants to inactivate tobamoviruses sorted by the object to be disinfected. In each case, the available information on the virus species, the inoculum, and the virus load, as well as the assessment method used, is provided.

Object	Virus	Type of Inoculum	Inoculum Load	Type of Bioassay	Assessment Type	Reference
clothing	ToBRFV	Infected *N. clevelandii* leaves	(1:5, *w*/*v*)	Local lesion	Semi-quantitative	[56]
Greenhouse surfaces	ToBRFV	Infected tomato leaves	(1:5, *w*/*v*)	Local lesion	Qualitative	[54]
	CGMMV	Contaminated surface	unknown	Systemic	Qualitative	[57]
	ToBRFV	Virus particles in non-infected Plant sap	1 mg/mL	Local lesion	Quantitative	[25]
Plant sap	TMV, ToMV	Infected tomato leaves	(1:5, *w*/*v*)	Systemic	Qualitative	[58]
	ToBRFV, CGMMV		(1:5, *w*/*v*)			[59]
	ToBRFV		(1:10, *w*/*v*)			[60]
Plant sap, knives, trays	CGMMV	Infected cucumber leaves	(1:5, *w*/*v*)	Systemic	Qualitative	[61]
Seeds	ToBRFV	Seeds from infected plant	Unknown	Systemic	Qualitative	[62]
	ToBRFV			Local lesion	Qualitative	[63]
	CGMMV			Systemic	Qualitative	[64]
Shoes	ToBRFV	infected *N. clevelandii* leaves	(1:5, *w*/*v*), (1:10, *w*/*v*)	Local lesion	Semi-quantitative	[65]
Soil	ToBRFV, CGMMV	Infected tomato/cucumber leaves	(1:5, *w*/*v*)	Systemic	Qualitative	[66]
	CGMMV	Infected dry *N. benthamiana* leaves	(1:20, *w*/*v*)	Systemic	Qualitative	[67]

ToBRFV: tomato brown rugose fruit virus; CGMMV: cucumber green mottle mosaic virus; ToMV: tomato mosaic virus.

## Data Availability

All data are shown in the text.
